# C-Type Natriuretic Peptide Plays an Anti-Inflammatory Role in Rat Epididymitis Induced by *UPEC*

**DOI:** 10.3389/fcimb.2021.711842

**Published:** 2021-08-12

**Authors:** Chunlei Mei, Yafei Kang, Chenlu Zhang, Chunyu He, Aihua Liao, Donghui Huang

**Affiliations:** ^1^Institute of Reproduction Health Research, Tongji Medical College, Huazhong University of Science and Technology, Wuhan, China; ^2^Reproductive Center, Jiangxi Maternal and Child Health Hospital, Nanchang, China

**Keywords:** C-type natriuretic peptide, epididymitis, uropathogenic *Escherichia coli*, cyclic guanosine monophosphate, protein kinases G, nuclear factor-k-gene binding

## Abstract

Human epididymitis is mainly caused by retrograde urinary tract infection with *uropathogenic Escherichia coli* (*UPEC*). This disease is an important factor (accounting for 20–30%) causing male infertility. C-type natriuretic peptide (CNP), a protein composed of 22 amino acids, is proved to play an immunoregulatory role in respiratory and cardiovascular systems. CNP is expressed extremely high in the epididymis, but whether CNP plays the same role in acute epididymitis is unclear. At first, we established an acute caput epididymitis model in rats with *UPEC* and treated them with CNP to measure inflammatory damage. Then RNA-seq transcriptome technology was used to reveal potential signal pathways. Secondly, the turbidity and activity of *UPEC* were assessed using a microplate reader and the amount of *UPEC* by agar plates after incubation with CNP. Thirdly, macrophages in caput epididymis were tested by immunohistochemistry (IHC). Meanwhile, lipopolysaccharide (LPS) with or without CNP was used to stimulate the macrophage (RAW264.7) *in vitro* and to detect the expression level of pro-inflammatory factors. Finally, the macrophage (RAW264.7) was treated with CNP, 8-Br-cGMP [cyclic guanosinc monophosphate (cGMP) analog] and KT5823 [protein kinase G (PKG) inhibitor], and the expression level of nuclear factor-k-gene binding (NF-kB) signal pathway was examined. The results showed that the damage of epididymis induced by *UPEC* as well as the pro-inflammatory factors could be alleviated significantly with CNP treatment. CNP could inhibit the activity and numbers of bacteria in both *in vivo* and *in vitro* experiments. Moreover, CNP repressed the invasion, and the expression of pro-inflammatory factors (such as NF-kB, IL-1β, IL-6, TNF-α) in macrophages and its effect could be inhibited by KT5823. Therefore, we drew a conclusion from the above experiments that CNP alleviates the acute epididymitis injury induced by *UPEC*. On one hand, CNP could inhibit the growth of *UPEC.* On the other hand, CNP could decrease invasion and inflammatory reaction of macrophages; the mechanism was involved in inhibiting NF-kB signal pathway through the cGMP/PKG in macrophages. This research would open up the possibility of using CNP as a potential treatment for epididymitis.

## Introduction

The epididymis is a long single highly convoluted tubule and is divided into four regions: initial segment, caput, corpus, and cauda (Stammler et al., 2015). The initial segment is mainly responsible for the absorption of testicular fluid, the caput is the key place where sperm maturation, while the cauda part is in charge of sperm storage (James et al., 2020). It is also an important site for sperm small RNA transportation, which is key to embryonic development (Conine et al., 2018). When the structure or function of epididymis is abnormal, it will lead to disorders in sperm transport or maturation, which can contribute to male infertility (Sullivan and Mieusset, 2016).

Epididymitis, a common intra-scrotal disease, is mainly caused by bacterial infection, particularly for *UPEC* (Frungieri et al., 2018). It has an incidence of 25–65/10,000 in adult males (Fijak et al., 2018) and mainly exists in sexually active people of 15–35 years old (Trojian et al., 2009). Moreover, the disease also occurs in children with an incidence of 1/1,000 (McConaghy and Panchal, 2016). The main signs and symptoms are scrotal redness, abdominal or groin pain, and epididymis swelling, fever, or other systemic infection, and so on (Lorenzo et al., 2016). Local inflammatory response in the epididymis eventually results in occlusion and fibrosis of the epididymal lumen, reducing sperm count and motility (Michel et al., 2016). Forty percent of patients would have persistent oligospermia or azoospermia (Michel et al., 2015) which may be due to immune response, intense fibrosis and epithelial cell damage (Centers for Disease et al., 2011) despite adequate antimicrobial treatment. Therefore, how to reduce local immune response or tissue damage is essential to avoid infertility caused by epididymitis.

CNP, a protein composed of 22 amino acids, is the third member of natriuretic peptide family (Sudoh et al., 1990). CNP is regarded as an autocrine and paracrine mediator released by endothelial cells to activate cGMP by binding to its specific receptor-natriuretic peptide receptor-B (NPRB or NPR2) and plays its biological role in local specific tissues (Sangaralingham and Burnett, 2018). Several studies showed that CNP in the male reproductive organ is much higher than that in other organs. CNP gene expression in pig epididymis is 125 times higher than that in heart, brain, and kidney (Nielsen et al., 2008). The concentration of CNP in porcine seminal plasma is 2,000 times higher than that in brain (Chrisman et al., 1993), whereas the protein level in human seminal plasma is 200 times that in plasma (Nielsen et al., 2005). It is demonstrated that CNP can increase cell proliferation, testosterone secretion in mouse Leydig cells through the receptor NPR2 (Yang et al., 2019), as well improve human sperm motility (Xia et al., 2016) and promote sperm capacitation through the cGMP/PKG pathway (Wu et al., 2019). In the female reproductive system, NPR2 is expressed not only in mouse oocytes (especially in germinal vesicle oocyte), but also in mouse embryos (especially in blastocyst stage) (Xi et al., 2019). Xia et al. further pointed out that the NPPC in granulosa cell ligand its receptor NPR2 in cumulus cells can prevent precocious meiotic maturation (Zhang et al., 2010), and this may be involved in FSH-mediated oocyte meiotic resumption by the EGFR and MAPK3/1 signaling pathways (Yang et al., 2016), which are critical for normal female fertility.

It has been reported that CNP also plays an immunoregulatory role in respiratory and cardiovascular systems. CNP can inhibit significantly the invasion of macrophages into the vascular endothelium during the formation of atherosclerosis and downregulate the monocyte chemoattractant protein 1 and vascular cell adhesion molecule by the CNP-PDE3-cGMP-PKA-NF-kB signaling pathway (Aizawa et al., 2003). Day et al. (2018) found that IFN-induced pro-inflammatory genes in human endothelial cells are suppressed by CNP *via* the cGMP-mediated pathway. Additionally, CNP can also reduce the MCP-1 levels in the myocardium and serum of rats with acute myocarditis as well as inhibit infiltration of inflammatory macrophages (Obata et al., 2007). In the respiratory system, Murakami et al. (2004) suggested that CNP downregulated interleukin-1β (*IL-1β*) in pulmonary fibrosis induced by bleomycin and repressed the invasion of macrophages and neutrophils. So, we wondered if CNP has similar anti-inflammatory effects on the male reproductive system.

Therefore, in this study, we aimed to study the anti-inflammatory effect of CNP on epididymitis and its possible mechanism, providing a potential treatment for epididymitis.

## Materials and Methods

### Rat Epididymitis Model

A rat acute caput epididymitis model was established under the guidance from Michel et al. (2016) and *UPEC *(ATCC25922)were donated by the lab of Professor Zhang Huiping, Huazhong University of Science and Technology. Adult male SD rats (weighing at 300–350 g) purchased from Hubei Bayonet Biotechnology were kept under suitable conditions and were randomly divided into three groups(five rats in per group): Control group, *UPEC* group, and *UPEC* + CNP group. Before 2 h of starting the model, the rats in the *UPEC* + CNP group were treated with intraperitoneal injection of CNP (50 ug/kg), while the rats in other groups were treated with equal volume of PBS. Next, these rats were anesthetized and incised in the lower abdomen. The right caput epididymis in the Control group was injected with 100 µl PBS, whereas rats in the other two groups were injected with 100 µl *UPEC* suspension (including about 10^6^ CFU). After the wound was sutured, these rats were kept warm until recovery. The rats in the *UPEC* + CNP group continued to receive the consecutive CNP for 2 days, while the other two groups received equal volumes of PBS. After 72 h, all rats were weighed and sacrificed, and then epididymal tissues were collected to weight and calculate the epididymal index (double epididymis weight/body weight * 100%) (Ward et al., 2001; Wang et al., 2014). Then, we collected caput epididymis of each rat. The entire experiment was repeated twice. The animal protocol was approved by the Ethics Committee of Animal Center of Tongji Medical College, Huazhong University of Science and Technology (No 2019S934).

### Histological Analysis

One part of caput epididymis tissue was sliced in a regular way for paraffin embedding and dewaxing later.

#### H&E Staining

The tissue slice was put into a hematoxylin solution (G1005,Servicebio, Wuhan, Hubei province, China) for a few minutes followed by acidic and ammonia water for a few seconds each before washing with running water. The next process is dehydration and eosin staining. The final stages were alcohol dehydration, xylene transparency, covering and observation with light microscopy (Nikon Eclipse E 100, Japan).

#### IHC

After the dewaxed sliced tissue was repaired with antigen and endogenous peroxidase was removed, the tissues were blocked for 1–2 h with a block solution (10% sheep serum + 5% BSA + PBS) at room temperature and washed with PBS. Then, the slices were incubated with CD68 macrophage antibody 1:800 (ab31630, Abcam) at 4°C overnight. Slices were washed thrice with PBS on the following day. Then, secondary antibody 1:200 (AS1106, ASPEN) was added and incubated for 1–2 h at room temperature followed by DAB staining (AR1022, BOSTER). When dyed brown, the slices were washed with running water to terminate DAB staining before hematoxylin dyeing for approximately 60–90 s. The final stages were alcohol dehydration, xylene transparency, covering and observation with light microscopy.

### Culturing and Counting the *UPEC* Bacteria

Part of the fresh bacterial suspension was placed into a sterile tube with LB culture solution. Then the tube was placed in a table concentrator at 37°C 220 rpm overnight. Part of the final *UPEC* suspension was used to establish animal model, but another part was diluted to 10^6^ CFU/100 µl for the *UPEC* incubation experiment which encompassed the following groups: ① Control (only LB culture solution); ② *UPEC*; ③ *UPEC* + CNP 10^−5^ mol/L; ④ *UPEC* + CNP 10^−6^ mol/L; ⑤ *UPEC* + CNP 10^−7^mol/L; ⑥ *UPEC* + Levofloxacin. A 100 µl *UPEC* suspension, different concentrations of CNP, and an equal amount of levofloxacin were added. After incubation for 6 h, optical density of ~0.1–1 at 600 nm is used for estimating bacterial concentrations with a microplate reader (AYNERGY HTX Gene Company), whereas bacterial activity was detected at 450 nm. Additionally, the amount of *UPEC* was calculated. The entire experiment was repeated thrice.

### RAW264.7 Cell Culture

The macrophage line RAW264.7 was replicated in culture medium (consisting of 90% 1640 culture medium, 10% fetal bovine serum, and 1% penicillin/streptomycin) in a cell incubator at 37°C and 5% CO_2_. Cells were seeded in six-well plate (about 6 × 10^4^ cells/well). We set three groups (each group had three repeated wells): ① Control (only cell suspension); ② LPS (100 ng/ml); ③ LPS + CNP 10^−7^ mol/L, and the cells in group ③ were pretreated with CNP for 30 min. After 6, 12, 16, and 24 h, all cell supernatants and cell precipitates were collected. However, there were six groups in another cell experiment which were incubated for 6 h: ① Control (only cell suspension); ② LPS (100 ng/ml); ③ LPS + CNP 10^−7^ mol/L; ④ LPS + 8-Br-cGMP 10^−4^ mol/L;⑤ LPS + CNP + KT5823 10^−6^ mol/L; ⑥ LPS + 8-Br-cGMP + KT5823. The cells were pretreated with CNP or 8-Br-cGMP or KT5823 for 30 min, respectively. The cell supernatant was used for ELISA, and cell precipitation was used for qPCR or Western blot.

### qPCR Assays and RNA-Sq Transcriptome Technology

#### Total RNA Isolation

Total RNA isolation was performed in a regular way with TRIzol-Chloroform. Finally, part of the extracted total RNA from the rat caput epididymis was used for reverse transcription. Another part was sent to Wuhan KangCe Technology Company for RNA-Sq transcriptome technology, and the sequencing results were again verified by qPCR.

#### Reverse Transcription

The steps were performed according to the instructions of a reverse transcription kit from Vzayme Company. The final cDNA product was immediately used for qPCR.

#### qPCR

The protocol followed the instruction of Hieff TM qPCR SYBR Green Master Mix (High ROX) provided by Yisheng Biotechnology Company. The volume of every well was 20 µl, which consists of 10 µl Hieff TM qPCR SYBR Green, 1 µl primer (forward primer 0.5 µl and reverse primer 0.5 µl), cDNA, and RNase-free water. Each sample was replicated thrice. All wells were placed into StepOne Plus TM Real-time PCR (Life Technologies Holding Pte. Ltd) to be heated. The parameters were the following: pre-denaturation at 95°C for 5 min, denaturation at 95°C for 10 s, annealing at 60°C for 20 s, extension at 72°C for 20 s for 40 cycles. The relative expression of target gene was standardized by b-actin, and the final gene expression was calculated with 2^-ΔCT^. The primer information of all revolved genes can be seen in **Table 1**.

**Table 1:** The primer sequence information of all involved genes.

Gene	Name	Rat	Mouse
*IL-6*	*Interleukin 6*	*F: CCACCCACAACAGACCAGTA* *R: CGGAACTCCAGAAGACCAGAG*	*F: GGCGGATCGGATGTTGTGAT* *R: GGACCCCAGACAATCGGTTG*
*TNF-α*	*Tumor necrosis factor*	*F: GTCGTAGCAAACCACCAAGC* *R: TCCCTCAGGGGTGTCCTTAG*	*F: CCTGTAGCCCACGTCGTAG* *R: GGGAGTAGACAAGGTACAACCC*
*IL-1β*	*Interleukin1 beta*	*F: CGTGGGATGATGACGACCTG* *R: GCCACAGGGATTTTGTCGTT*	*F: GAAATGCCACCTTTTGACAGTG* *R: TGGATGCTCTCATCAGGACAG*
*b-actin*	*Beta-actin*	*F: GAAGGTCGGTGTGAACGGAT* *R: CCCATTTGATGTTAGCGGGAT*	*F: AATGGATTTGGACGCATTGGT* *R: TTTGCACTGGTACGTGTTGAT*
*PapC*	*Outer membrane usher protein*	*F:GACGGCTGTACTGCAGGTGTGGCG* *(UPEC)*	*R: ATATCCTTTCTGCAGGGATGCAATA* *(UPEC)*
*Fgb*	*Fibrinogen beta chain*	*F: CACCGTCAACTGCAACATCC* *R: TATGACCGTCCATCCTCCGT*	
*Gtsf1*	*Gametocyte specific factor 1*	*F: CAGTGCCCTCCTTGTGATGA* *R: TTTGCAGGACTGTTGTTGCC*	
*Gata4*	*GATA binding protein 4*	*F: ATGGGTCCTCCATCCATCCA* *R: GCTGTTCCAAGAGTCCTGCT*	
*Mmp8*	*Metal matrix protein*	*F: TCTCTGTTCTGGCCCTTCCT* *R: GCTGCATCAATGGCTTGGAC*	
*Alox5ap*	*Arachidonate 5-lipoxygenasea*	*F: GCCAACCAGAACTGCGTAGA* *R: CAGATACATCAGCCCAGCGA*	
*Ncf1*	*Neutrophil cytosolic factor 1*	*F: GGACACCTTCATTCGCCACAT* *R: TGTAGACCACCTTCTCCGACA*	
*Defb1*	*Defensin beta 1*	*F: CACTCTGGACCCTGACTTCAC* *R: TCTGTTCTGCGTCCAAGACT*	
*Gbp1*	*Guanylate binding protein 1*	*F: GATGGAGAGGGACAGAGCAC* *R: GATGGAGAGGGACAGAGCAC*	
*Bin1b* *(Spag11a)*	*Sperm associated antigen 11a*	*F: AGTCTCATCTGCTTTCCTGCAC* *R: CACGGTGTTTCTGATTCCAGG*	

### Cell Viability Assays

The instructions of the CCK-8 kit (DOJINDO, Shanghai Yisheng Biotechnology Company) were followed for the operation. RAW264.7 cell suspensions were plated in 96-well plates (approximately 5,000 cells/well). The experiment set the following groups (each group had five repeated wells): ① Blank (no cells); ② Control (only cell suspension); ③ Lipopolysaccharide (LPS 100 ng/ml); ④ LPS + CNP 10^−7^ mol/L. After incubation for 6, 12, 16, and 24 h, the supernatant was removed, and the well was washed with PBS thrice. Then, 100 µl CCK-8 solution diluted with cell culture medium was added, and cells were incubated to produce a visible brown color. Finally, the cell activity was detected by a microplate reader at 450 nm.

### Western Blot Analysis

After the total protein of rat caput epididymis tissues and cell precipitate were extracted and prepared, the constant pressure electrophoresis was performed in a concentrating gel at 80 V followed by a separating gel at 120 V until bromophenol blue reached the bottom edge of the gel. Then, the protein was electrophoretically transferred to 0.44 µM PVDF membranes (Millipore, Bedford, MA, USA) at 300 mA for 60 min on ice. The non-specific binding sites on the membrane were blocked with 5% fat-free milk at room temperature for 2 h. Next, the membrane was incubated with primary antibody NF-kB P65 1:1,000 (EM1111, ELK Biotechnology) at 4°C overnight and developed with a second antibody 1:10,000 (AS1106, ASPEN) at room temperature for 30–60 min. Then, the membrane was washed four times with TBST, and ECL mixed solution was added onto the membrane to be visualized using enhanced chemiluminescence luminol reagent (PerkinElmer Inc, Boston).

### ELISA

Interleukin-6 (IL-6), tumor necrosis factor (TNF-α), *IL-1β*, and cGMP ELISA kits were purchased from Tianjin Anoric Biotechnology Company, and CNP ELISA kit was purchased from BlueGene Company. All operations were performed according to the instructions of the corresponding ELISA kits. Equal volumes of standard sample and tested sample were added to 96 wells in sequence (every sample had three repeated wells). Sequentially, equal volumes of biotin antibodies and horseradish peroxidase-labeled antibody were added to the tested sample wells and then incubated at 37°C. After washing all wells, color liquid was added to incubate under no light. Finally, stop buffer was added and detected using a microplate reader at 450 nm.

### Immunofluorescence

RAW 264.7 cell suspensions were plated onto glass coverslips in six-well plates. After 15 h, the glass coverslips were fixed with 4% PFA (Biosharp, Beijing, BL539A) for 20 min and washed with PBS three times for 5 min each time. The autofluorescence quenching agent was added for 5 min. Then the slides were rinsed for 10 min and incubated with 3% hydrogen peroxide for 30 min. After that, the slides were incubated with anti-NPR-B antibody (Santa, 1:200) overnight at 4°C followed by incubation with a secondary FITC Rabbit Anti-goat IgG for 50 min at room temperature. Nuclei were stained with DAPI for 10min.

### Statistical Analysis

We utilized GraphPad Prism 6 software to analyze the data, and P-value less than 0.05 was defined as statistically significant, *p < 0.05, **p < 0.01, ***p < 0.001. All data were analyzed with parametric tests.

## Results

### CNP Alleviates Epididymal Damage in Epididymitis

**Figure 1:**
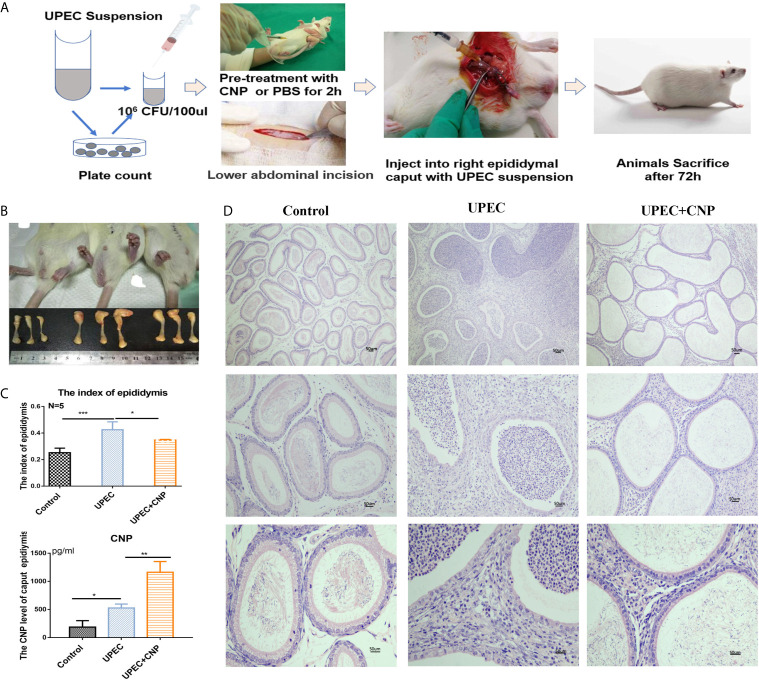
Morphological traits of acute caput epididymitis in rats. **(A)** Steps to establish the model of epididymitis. **(B)** Visual changes of caput epididymitis and rat epididymal index. **(C)** The CNP content of caput epididymal fluid was determined by ELISA. **(D)** H&E staining showed the characteristics of rat caput epididymitis under a microscope. Bar = 50 um. The data shown are representative of multiple experiments (mean ± SD, n = 5, *P < 0.05, **P < 0.01, ***P < 0.001).

A rat acute caput epididymitis model was established as shown in **Figure 1A**. The rats exhibited redness and swelling in the scrotum and the caput epididymis was obviously red and swollen. The inflammatory reaction and the epididymal index in the *UPEC* + CNP group were obviously lower than those in the *UPEC* group (*P* < 0.05) (**Figures 1A, B**). Then, the result of CNP content in the rat caput epididymal fluid exhibited that the protein in the *UPEC* + CNP group was more than that in the other groups (*P* < 0.05) (**Figure 1C**), indicating that CNP could accumulate and play a role in the caput epididymis after intraperitoneal injection. H&E staining showed that epididymal lumen and stroma were filled with a large number of pro-inflammatory cells and fibroblasts; the epididymal epithelial cells collapsed and arranged disorderly, and there were no normal sperms in the lumen in the *UPEC* group (**Figure 1D**). This damage was less severe after CNP intervention (**Figure 1D**).

### The Features of Caput Epididymal Genes Sequenced by RNA-Seq Transcriptome Technology

**Figure 2:**
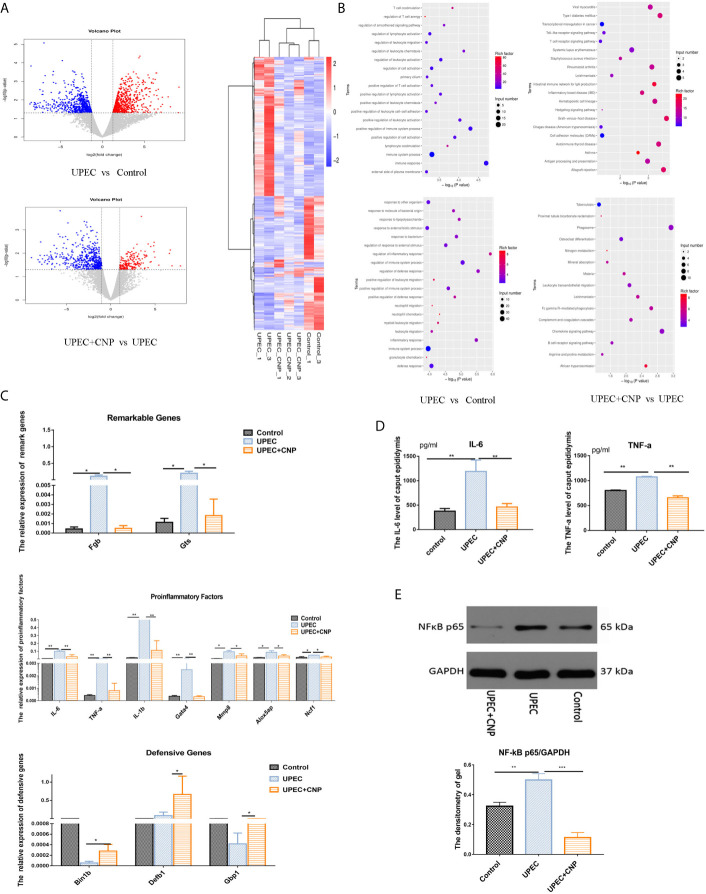
RNA-seq sequencing and verification of genes. **(A)** Volcano graph and heatmap; blue represents downregulation, and red represents upregulation. **(B)** GO analysis and KEGG analysis between each group. **(C)** Gene verification with qPCR. **(D)** The content of IL-6 and TNF-a in caput epididymal fluid. **(E)** Expression level of NF-kB protein in caput epididymal tissue and was quantified in images. (mean ± SD, n = 3, *P < 0.05, **P < 0.01, ***P < 0.001).

According to the volcano graph, 666 genes were upregulated and 625 genes were downregulated in the *UPEC* group compared with the Control group; 95 genes were upregulated and 304 genes were downregulated in *UPEC* + CNP group compared with the *UPEC* group (**Figure 2A**). These genetic changes were mapped into a heatmap (**Figure 2A**). GO analysis showed the involved responses of these genes, including pro-inflammatory responses to bacteria, leukocyte migration and innate immune responses, *etc*. (**Figure 2B**). Meanwhile, KEGG analysis explained relevant signaling pathways, such as the *TNF-α* signaling pathway and NF-kB signaling pathway (**Figure 2B**).

To verify RNA-seq sequencing results, we chose two significantly different genes (*Fgb* and *Gtsf1*), seven pro-inflammatory genes (*IL-6*, *TNF-α*, *IL-1β*, *Gata4*, *Mmp8*, *Alox5ap*, and *Ncf1*) and three genes associated with defense reactions (*Bin1b*, *Defb1*, and *GBP1*) (**Figure 2C**) to be tested again with qPCR. The results showed that seven pro-inflammatory genes in the *UPEC* group increased, and levels of defensive genes in the *UPEC* + CNP group increased compared with those in the *UPEC* group (*P* < 0.05). Furthermore, the protein expression of pro-inflammatory factors (such as IL-6, TNF-α, NF-kB) in caput epididymal fluid (**Figure 2D**) and the protein was detected by Western blot and was quantified in images (**Figure 2E**). The two results showed that these inflammation factors were more abundant in the *UPEC* group than in the Control group (*P*<0.05), whereas CNP reduced the expression of inflammatory factors. Therefore, it can be hypothesized that CNP may demonstrate its function by inhibiting the NF-kB pathway and promoting defensive genes.

### CNP Suppresses *UPEC In Vivo* and *In Vitro*

**Figure 3:**
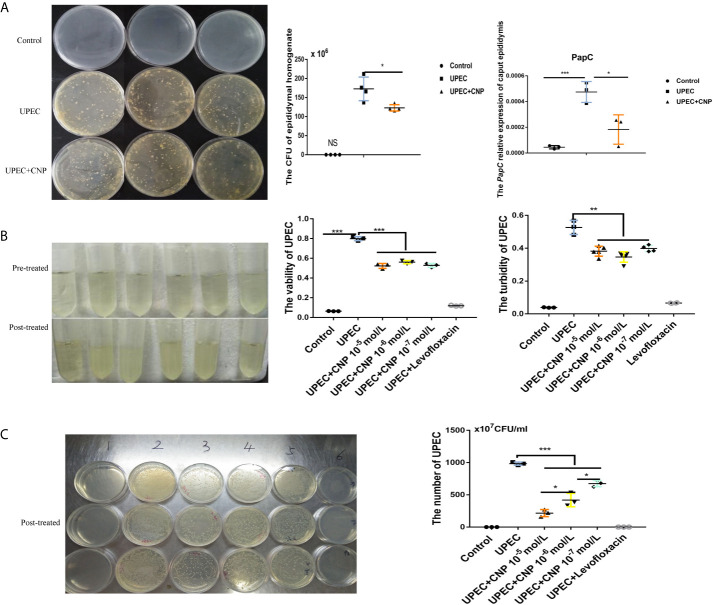
CNP inhibits proliferation and activity of *UPEC* bacteria *in vivo* and *in vitro*. **(A)** The number of bacteria in rat caput epididymal fluid and the level of *PapC* mRNA in rat caput epididymis. **(B)** The turbidity of bacterial suspension and *UPEC* activity after incubation for 6 h *in vitro*. **(C)** The number of *UPEC* after incubation for 6 h *in vitro*. The data shown are representative of multiple experiments (mean ± SD, n = 3, *P < 0.05, **P < 0.01, ***P < 0.001). NS, no sample.

In *in vivo* experiment, the *PapC* gene is a flagellum gene of *UPEC*, which can be used to assess *UPEC* load. The results demonstrated that the bacterial number was numerous and the level of *PapC* gene (**Figure 3A**) in the *UPEC* group was high. *In vitro*, the bacterial turbidity of the CNP intervention group exhibited a significant decrease compared with that of other groups (*P* < 0.01), and the bacterial activity also showed a similar trend (*P* < 0.001) (**Figure 3B**). Furthermore, the number of bacteria was lower after CNP incubation (**Figure 3C**).

### CNP Down-Regulates Pro-Inflammatory Factors by Affecting the Infiltration and Activity of Macrophages

**Figure 4:**
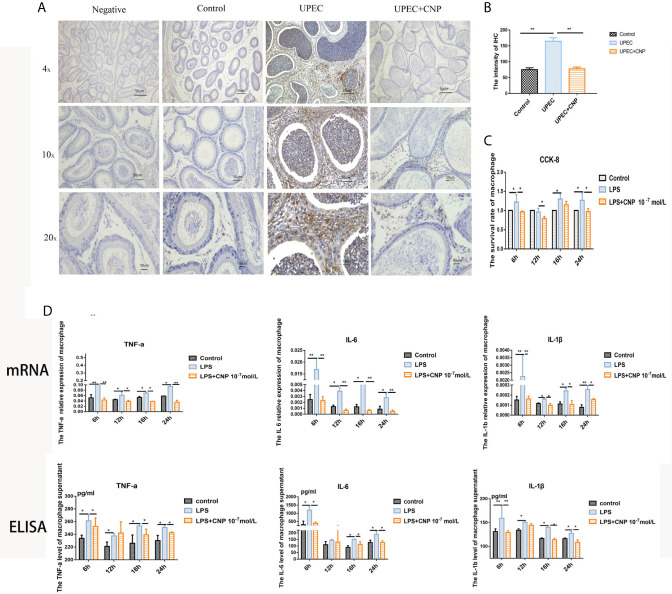
CNP downregulates pro-inflammatory factors by affecting the infiltration and proliferation of macrophages. **(A)** IHC to detect macrophages in caput epididymis. **(B)** Histogram showed the degree of macrophage infiltration. **(C)** CCK-8 assay of the proliferative activity of inflammatory macrophages. **(D)** mRNA levels and concentrations of *IL-6*, *TNF-α*, and *IL-1β*. The data shown are representative of multiple experiments (mean ± SD, n = 3, *P < 0.05, **P < 0.01).

*In vivo*, IHC revealed that the epididymal stroma and the lumen were both filled with a large number of macrophages quantified in images, and the epididymal epithelial cells were destroyed and fell off in the *UPEC* group (**Figures 4A, B**). However, the phenomenon was improved in the *UPEC* + CNP group (**Figures 4A, B**). Furthermore, *in vitro*, the CCK-8 results indicated that CNP reduced the proliferation activity of inflammatory macrophages line Raw264.7 (*P* < 0.05) (**Figure 4C**). qPCR detection (**Figure 4C**) showed that the mRNA levels as well as the concentrations of *IL-6*, *TNF-α*, and *IL-1β* released by macrophages in the LPS + CNP group (**Figure 4D**) decreased. These results suggested that CNP represses the secretion of pro-inflammatory factors by affecting the infiltration and proliferation of inflammatory macrophages.

### CNP Represses NF-kB to Release Pro-Inflammatory Factors by the cGMP/PKG Signaling Pathway in Macrophages

**Figure 5:**
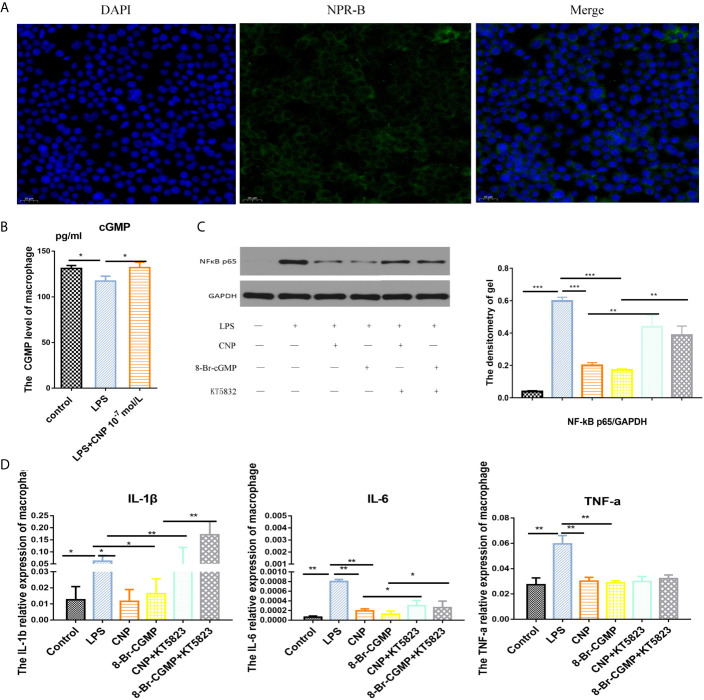
CNP represses NF-kB to release pro-inflammatory factors by the cGMP/PKG signaling pathway in macrophages. **(A)** Immunofluorescence showed the location of NPR-B in RAW264.7macrophages. **(B)** ELISA revealed intracellular cGMP level in macrophages cultured with CNP for 6 h. **(C)** Western blot analysis showed NF-kB protein expression after incubation with CNP, 8-Br-cGMP or KT5823 alone or in combination and was quantified in images. **(D)** qPCR showed the mRNA levels of the downstream pro-inflammatory cytokines *IL-6*, *TNF-α*, and *IL-1β*. The data shown are representative of multiple experiments (mean ± SD, n = 3, *P < 0.05, **P < 0.01, ***P < 0.001).

Immunofluorescence confirmed NPR-B was expressed in RAW264.7macrophages (**Figure 5A**), and the concentration of cGMP in macrophages increased after incubation with CNP (**Figure 5B**). Western blot result showed that NF-kB expression in the LPS group was decreased significantly after the addition of CNP and 8-Br-cGMP. The KT5823 reversed the inhibitory effect of CNP on NF-kB expression (**Figure 5C**). Additionally, the gene levels (**Figure 5D**) of the downstream *IL-6*, *TNF-α*, and *IL-1β* kept the same trend as that of *NF-kB*, confirming that the signaling pathway was feasible.

## Discussion

Our study demonstrated that CNP can significantly alleviate the inflammatory reaction and tissue injury of acute epididymitis. On the one hand, CNP can inhibit the proliferation and activity of *UPEC* and has antibacterial effect. On the other hand, CNP can inhibit the invasion of macrophages and reduce their secretion of pro-inflammatory factors by cGMP-PKG-NF-kB pathway. Therefore, this study confirmed for the first time that CNP has anti-inflammatory and immunomodulatory effects on acute epididymitis, and its mechanism was revealed.

Currently, epididymal immunological regulation mainly depends on the following four aspects: ① Blood–epididymal barrier (Franca et al., 2012): Sperm can avoid immune attack through the barrier. Infection can change or destroy the barrier, causing immune response and sperm function damage (Fijak et al., 2018). ② Mononuclear macrophage system: such as dendritic cells and macrophages. The former mainly exist in the epididymal epithelial basal area and expresses antigen-presenting surface molecules (Da Silva and Barton, 2016). The latter are the main immune cells in the epididymis. If infected by *UPEC*, they would bind toll-like receptor 4 or toll-like receptor 5 to activate NF-kB signaling pathways and stimulate the secretion of pro-inflammatory factors (Cheng et al., 2016). ③ Lymphocytes: CD8+ T cells are main lymphocytes in the epididymal epithelium, whereas B lymphocytes are rare (Voisin et al., 2018). ④ Immunomodulatory factors: Some immunomodulatory factors have been found to be highly expressed in the caput epididymis, such as indoleamine 2,3-dioxygenase, activin A, and Bin1b (Li et al., 2001), which not only can promote the tolerance of the epididymis to sperm antigens, but also can resist pathogen infection (Wijayarathna and Hedger, 2019). In this study, we found that CNP relieved the redness and swelling in the scrotum and reduced the secretion of inflammatory factors in epididymitis. The results suggest that CNP is involved in the immunoregulation of epididymitis. But the mechanism is not clear. To date, only one related study indicated the relationship between CNP and epididymitis, which found that the concentration of CNP in the seminal plasma of azoospermia patients was significantly increased compared with that of normal individual, which may be due to urogenital tract inflammation, such as epididymitis (Tomasiuk et al., 2017). However, this study did not investigate the role of CNP in epididymitis.

It is acknowledged that human epididymitis is mainly caused by retrograde urinary tract infection with *UPEC* (Frungieri et al., 2018). Cheng et al. (2016) found that *UPEC* infection in the epididymis could increase *IL-6* and *TNF-α* through NF-kB signaling by combining with toll-like receptor 4 or toll-like receptor 5. Wang et al. (2019) used transgenic mice to also show that TNF-α was the critical inflammatory factor in the epididymitis. Cao et al. (2010) revealed that LPS can bind TLR4 to induce an inflammatory cascade reaction, which stimulates myeloid differentiation factor 88 expression and downstream NF-kB entry into nucleus. RNA-seq analysis of caput epididymitis induced by LPS showed it mainly upregulates inflammation related genes (such as TNF-a, heat shock protein) and activates the signal pathways (NF-kB, MAPK, *etc*.) in epididymis (Song et al., 2019). In our *UPEC*-induced rat epididymitis model, CNP could significantly alleviate the levels of pro-inflammatory related genes (such as *IL-6*, *TNF-a*, *IL-1β*, *Gata4*, *Mmp8*, *Alox5ap*, *Ncf1*) and promote some defense genes (*Bin1b*, *Defb1*, *Gbp1*, *etc*). GO analysis and KEGG analysis showed that the related mechanisms involve leukocyte migration, cytokine response, TNF-a signaling pathway, NF-kB signaling pathway, NOD-like receptor signaling pathway, *etc*.

Pathogen infection not can only lead to immune response, but also can directly lead to inflammatory injury. Studies have demonstrated that incubation of human sperm directly with *UPEC* can reduce mitochondrial potential and may increase oxidative damage of sperm (Boguen et al., 2015). Moreover, *UPEC* can enter epithelial cells of the urogenital tract and migrate to obtain nutrients and avoid killing (Lewis et al., 2016). Quinolone drugs, such as levofloxacin, can prevent bacterial DNA synthesis and replication, resulting in bacterial death (Klein et al., 2019). Britta et al. used levofloxacin combined with glucocorticoid to treat epididymitis in mice, and found it significantly reduced the inflammatory cell infiltration of epididymitis and the bacterial load of *UPEC* compared with antibiotics alone, and the reason was that glucocorticoid inhibited the pro-inflammatory response of immune cells (Klein et al., 2019). Maria et al. demonstrated the mechanism as follows: glucocorticoid can bind with the glucocorticoid receptor of epididymal epithelium, and then form a complex with heat shock protein 90 (Hsp90), resulting in the increased expression of Ikba kinase, limiting the nuclear expression of NF-kB (Silva et al., 2011). In this study, the number of bacteria and the mRNA level of *PapC* decreased after CNP intervention *in vivo*. *In vitro*, CNP can directly repress *UPEC* to play a protective role. However, the antibacterial mechanism remains unclear and needs to be further explored.

Macrophages, a major phagocyte, play an important role in host immune defense and regulation of inflammatory response. They are the most abundant immune cells in epididymis (Michel et al., 2015) and chiefly in the interstitial and peritubular regions (Shum et al., 2014). Several data showed that CNP could inhibit macrophage infiltration *in vivo*. Itoh et al. (2004) demonstrated that CNP inhibits the local infiltration of macrophages in rat pulmonary hypertension. The overexpression of CNP in fat cells suppresses the infiltration of macrophages in the mesenteric fat pad to reduce the inflammatory response (Bae et al., 2018). Kiemer and Vollmar (1997) reported that RAW264.7 macrophages expressed NPR-B which can also be found in human macrophage (Baldini et al., 2003). In our *UPEC*-induced rat epididymitis model, epididymal stroma and cavity were full of macrophages, while CNP dramatically reduced the number of macrophages, indicating that CNP can decrease the invasion of macrophages *in vivo*. We confirmed the expression of NPR-B in the RAW264.7 macrophages and found that CNP could inhibit the proliferation of RAW264.7 macrophages *in vitro*. Moreover, CNP can reduce the pro-inflammatory factors secretion of macrophages *in vitro*. These results suggest that CNP plays an anti-inflammatory role by inhibiting infiltration and activity of macrophages in epididymitis.

NF-kB is a dimer transcription factor from the Rel family and is a crucial regulator in many physiological and patho-physiological processes, including immunity, inflammation, cell proliferation, apoptosis, *etc*. (Mitchell et al., 2016). Many studies showed LPS could activate toll-like receptors on macrophages and promote the myeloid differentiation factor 88, which could induce NF-kB to enter the nucleus, and increase section of pro-inflammatory factors (*IL-6*, *TNF-a*, *IL-1β*, *etc*.) (Hobbs et al., 2018; Jin et al., 2019; Li et al., 2019). In LPS-induced RAW264.7 macrophages, we found that CNP significantly downregulated expression of NF-kB and pro-inflammatory factors, which indicates that CNP represses the NF-kB signal pathway in inflammation macrophages and plays anti-inflammation function.

cGMP is a second messenger molecule in intracellular information transmission, regulating a broad array of physiologic processes. The elevated intracellular cGMP level exerts its physiological action mainly through PKG. In the urinary system, cGMP/PKG can suppress macrophages replication to play a protective role (Zhang et al., 2017). Increased PKG activity also significantly inhibits the invasion of macrophages to decrease renal fibrosis (Cui et al., 2014). Overexpression of PKG prevents the infiltration of macrophages in renal ischemia–reperfusion (Li et al., 2012). On the other hand, the increase of PEG2 caused by carbon monoxide in macrophages may activate downstream PKG to downregulate *IL-1β* (Lin et al., 2010). The latest research indicates that cGMP has the potential to treat infertility in the epididymis (Zhang et al., 2020). In this experiment, CNP could increase level of cGMP in RAW264.7 macrophages, and its effect on NF-kB signal pathway was inhibited by PKG inhibitor KT5823. Therefore, CNP could downregulate NF-kB signal pathway in macrophage through the cGMP/PKG pathway.

**Figure 6:**
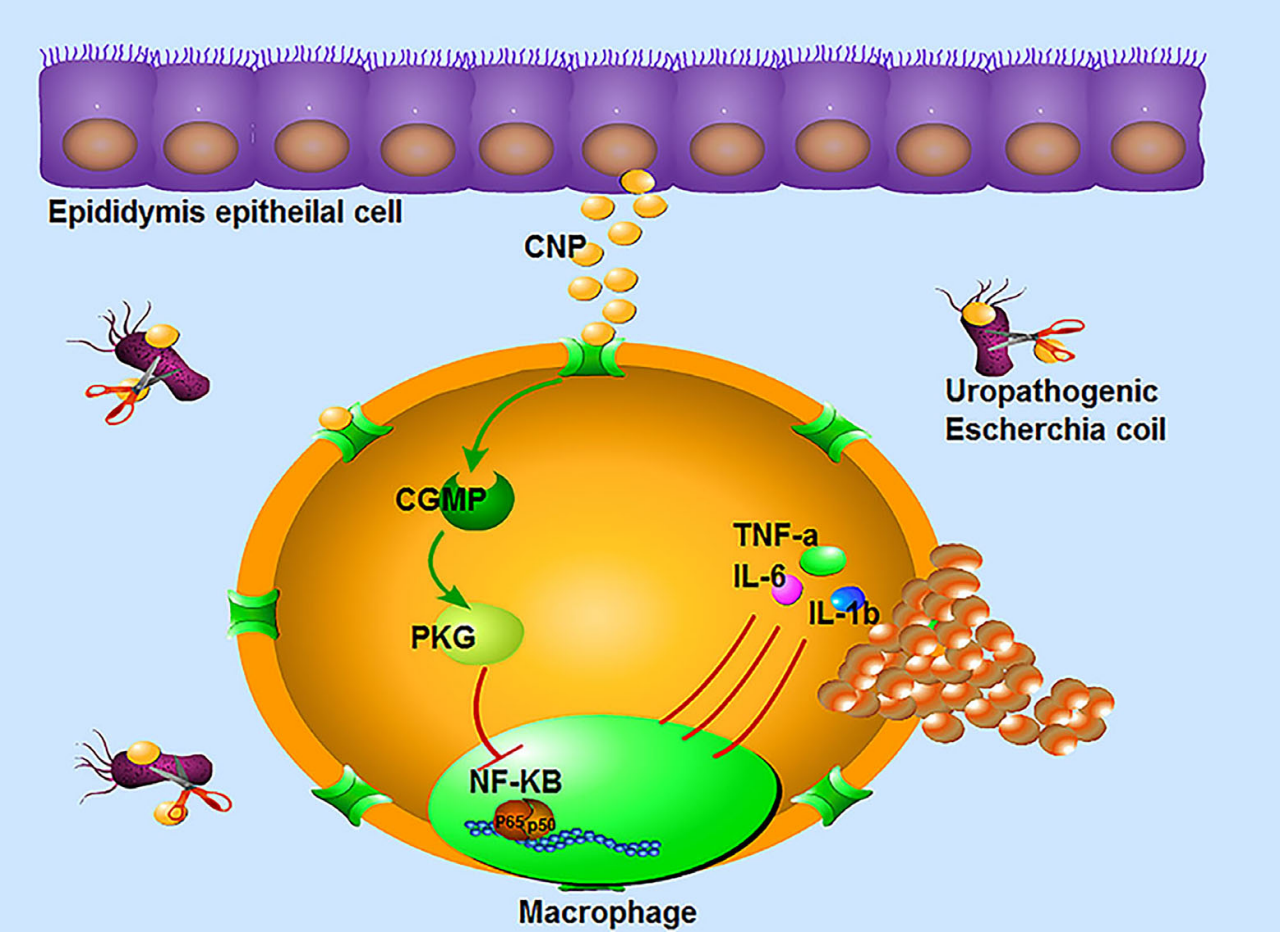
The immunomodulatory mechanism of CNP in epididymitis: ① CNP plays an antibacterial role by inhibiting the proliferation and activity of *UPEC*. ② CNP not only represses the infiltration, proliferation of macrophages, but also downregulates the secretion of pro-inflammatory factors by the cGMP-PKG-NF-kB signaling pathway in macrophages.

In conclusion, CNP can significantly reduce the injury in epididymitis. Regarding the relevant mechanism, on one hand, CNP inhibits *UPEC* growth. On the other hand, CNP affects the infiltration and proliferation of macrophages and downregulates NF-kB to secrete pro-inflammatory factors by the cGMP/PKG signaling pathway in macrophages (**Figure 6**). It can be inferred from the above data, CNP may become a potential treatment for epididymitis. This manuscript does open up the possibility of using a lower dose of antibiotics when combined with CNP.

## Data Availability Statement

The original contributions presented in the study are publicly available. This data can be found in the NCBI SRA (Sequence Read Achieve) database with the accession number of PRJNA749898.

## Ethics Statement

The animal study was reviewed and approved by Ethics Committee of Animal Center of Tongji Medical College, Huazhong University of Science and Technology(No 2019S934).

## Author Contributions

CM and YK performed experiments, and completed, analyzed data compilation, and wrote the article. CM and CH performed the necessary literature searches and rectified. AL revised the article and give valuable suggestions. DH designed the study, reviewed this manuscript. All authors contributed to the article and approved the submitted version.

## Funding

This work was supported by the National Natural Science Foundation of China (grant number NNSF 81771575), National Key Research & Developmental Program of China (2018YFC1003900) and the Independent Innovation Foundation of Tongji Medical College of Huazhong University of Science and Technology (grant number IIF 5003510033).

## Conflict of Interest

The authors declare that the research was conducted in the absence of any commercial or financial relationships that could be construed as a potential conflict of interest.

## Publisher’s Note

All claims expressed in this article are solely those of the authors and do not necessarily represent those of their affiliated organizations, or those of the publisher, the editors and the reviewers. Any product that may be evaluated in this article, or claim that may be made by its manufacturer, is not guaranteed or endorsed by the publisher.

## Abbreviations

CNP, C-type natriuretic peptide; *UPEC*, *Uropathogenic Escherichia coli*; HE, H&E staining; qPCR, Quantitative polymerase chain reaction; ELISA, Enzyme Linked Immunosorbent Assay; cGMP, Cyclic guanosinc monophosphate; PKG, Protein kinases G; LPS, Lipopolysaccharide; IL-6, Interleukin-6; TNF-α, Tumor Necrosis Factor; IL-1β, Interleukin-1β; NF-kB, Nuclear factor-k-gene binding.
